# Multiple criteria optimization joint analyses of microarray experiments in lung cancer: from existing microarray data to new knowledge

**DOI:** 10.1002/cam4.540

**Published:** 2015-10-16

**Authors:** Katia I. Camacho‐Cáceres, Juan C. Acevedo‐Díaz, Lynn M. Pérez‐Marty, Michael Ortiz, Juan Irizarry, Mauricio Cabrera‐Ríos, Clara E. Isaza

**Affiliations:** ^1^ Bio IE LabThe Applied Optimization GroupIndustrial Engineering DepartmentUniversity of Puerto RicoMayaguezPuerto Rico; ^2^ Public Health ProgramPonce Health Sciences UniversityPoncePuerto Rico

**Keywords:** Biomarker, lung cancer, meta‐analysis, multi‐criteria optimization

## Abstract

Microarrays can provide large amounts of data for genetic relative expression in illnesses of interest such as cancer in short time. These data, however, are stored and often times abandoned when new experimental technologies arrive. This work reexamines lung cancer microarray data with a novel multiple criteria optimization‐based strategy aiming to detect highly differentially expressed genes. This strategy does not require any adjustment of parameters by the user and is capable to handle multiple and incommensurate units across microarrays. In the analysis, groups of samples from patients with distinct smoking habits (never smoker, current smoker) and different gender are contrasted to elicit sets of highly differentially expressed genes, several of which are already associated to lung cancer and other types of cancer. The list of genes is provided with a discussion of their role in cancer, as well as the possible research directions for each of them.

## Introduction

According to the International Agency for Research on Cancer, the world's most commonly diagnosed cancer is lung cancer, with 1.8 million cases or 13% of total in 2012. Additionally, lung cancer was the first cause of death in the world, with 1.6 million deaths or 19.4% in 2012 1. This analysis was conducted in 184 countries. This work intends to facilitate uncovering new information related to cancer using publicly available lung cancer microarray data. The aim is to find those genes that changed their relative expression the most in order to propose potential lung cancer biomarkers.

Microarray experiments quantify the relative expression of tens of thousands of genes. These experiments have been highly utilized in the past decade to study a number of health conditions, including cancer 2, 3. These experiments, however, are often times measured in different units, thus making it difficult to analyze several of them simultaneously. Furthermore, because the measured level of expression is relative, a normalization process is commonly required. All of these have hampered the search for cancer biomarkers in the past.

The strategy to detect potential biomarkers utilized in this work is based on mathematical optimization. Optimization can be defined as a decision‐making process aimed to obtain the best possible values in a series of performance measures (PMs) of interest. The decision variables are habitually constrained to fall within specific ranges or to maintain mathematical relationships among them 4, 5. Mathematical optimization (MO) has been widely used in many fields, including Economics and Engineering, and clearly it can be applied to biological analysis. MO can make a system or design effective, functional, or in its most basic form, possible 6, 7. Multiple Criteria Optimization (MCO) is an optimization problem that finds a set of solutions corresponding to the best possible balances among two or more conflicting PMs under study 8. These solutions are known as Pareto‐Efficient solutions and are mathematically characterized by the well‐established Pareto‐optimality conditions. In general terms, then, the idea behind a MCO problem is to find the Pareto‐Efficient Frontier formed by the Pareto‐efficient solutions.

In this work the analysis of a publicly available microarray database for lung cancer is presented as a MCO problem. The genetic expression changes in this analysis were quantified using two metrics that do not have a perfect correlation and thus, are in conflict: difference of means and difference of medians. For the analysis, the MCO solutions will be those genes that have associated the greatest differences in the selected metrics. The solution genes are the ones that changed their expression the most between the compared conditions and could be potential biomarkers and can, after further study and confirmation, help with the diagnosis, prognosis, treatment, and recurrence prediction for the condition under analysis 9. It can be appreciated that the method seeks to minimize the likelihood of false positives due to its focus on frontier analysis. This, expectedly, comes at the cost of false negatives in genes that might not appear in the Pareto‐efficient frontier due to experimental error. A simple strategy of finding several consecutive frontiers is proposed to alleviate this issue.

The analysis strategy in this paper has, however, the advantage of providing objectivity, as it does not require the analyst to change or adjust any parameters, thereby fostering repeatability across analysts. It has also been shown to have a high discrimination power. The method used to solve the associated MCO problem is a full pairwise comparison scheme that effectively finds the genes that show high expression change across multiple PMs. This scheme is an improvement in terms of precision and convergence over the Data Envelopment Analysis approach presented by our group in 9. The genes identified this way are located on the Pareto‐efficient frontier of the MCO problem, that is, they are demonstrably Pareto‐efficient 10 and are, in consequence, proposed as potential biomarkers.

## Literature Review

Microarray experiments have been very popular among researchers 11. In the Gene Expression Omnibus (GEO) as of May 2015 there are 3848 databases with 1,392,278 samples. Microarray experiments are sufficiently accepted as a reliable technology where the most common use is to find differentially expressed genes between two experimental conditions or samples 12. Moreover, microarrays have been used to study how different biological processes or pathways work in several organisms 13. To analyze the experimental data, statistics have been used for these types of studies 14, 15. However, producing a standard method for analysis has never been accomplished.

In the literature there are many methods to find highly differentially expressed genes to characterize them as potential biomarkers. Most of them focus on statistical procedures 15, 16. This research adopts multiple criteria optimization and Pareto conditions to find biomarkers following the direction of our research group 9, 17, and proposes extending the application to this end through simultaneous analysis of multiple independent experiments, that is carrying out meta‐analysis. In 2010, in our group, Sanchez‐Peña 9 used a combination of two performance measures (two *P*‐values) obtained from a single‐microarray database to cast the MCO problem and Data Enveloped Analysis (DEA) to solve it. The pairwise comparison scheme in the present work yields a more precise Pareto‐efficient frontier than DEA, as it can deal with nonconvexity from the onset.

An important direction of this work is to use the proposed method for meta‐analysis of high throughput biological experiments, starting with microarrays. Glasser and Duval 18 provide the definition: “Meta‐analysis refers to methods for the systematic review of a set of individual studies or patients within each study, with the aim to quantitatively combine their results.” Meta‐analysis is a method capable of taking independent, but associated studies to obtain a set of solutions through all studies. It is possible to find different applications and examples about meta‐analysis. Li and his research group led a systematic review and meta‐analysis to determine whether two polymorphisms (V89L and A49T) are associated with the risk of prostate cancer. They found 31 articles and reviews related to such risk 19. On the other hand, R makes available a tool for microarray meta‐analysis called MetaOmics. MetaOmics integrates Quality Control (Meta QC), Differentially Expressed (Meta DE), and Pathway (Meta pathway) 20. Also, Zhuohui et al. (2014) research developed a tool, “MAAMD” 21. They carried out meta‐analysis using Affymetrix microarray data. The tool automates the process to analyze microarrays and requires normalization and several statistical methods to detect differential gene expression. To this end, they used Kepler, AltAnalyze and Bioconductor software packages. The parametric approaches in these works differ from our nonparametric approach. Therefore, it is clear that multiple criteria optimization differs from the reviewed approaches and constitute a novelty in meta‐analysis. It must be emphasized, however that meta‐analysis is a study that comprehends a larger area than afforded by the use of a single technique and that it requires a methodical design to be reliable. Especial care, for instance, must be given to the selection of studies to be included 22, as well as their heterogeneity 23. Meta‐analysis has become a cornerstone for evidence‐based medicine 24 and follows widely accepted standards for its realization 25, 26, 27.

As noted earlier, the MCO problem has been approached in our group by Sánchez‐Peña, et al. 9 through Data Envelopment Analysis (DEA). This work approaches the larger problem of analyzing multiple microarray databases simultaneously that is, to carry out meta‐analysis, formulating the analysis as an MCO problem and solving it through a pairwise‐comparison scheme that facilitates the evaluation of Pareto‐efficiency conditions. In the literature, the authors of 28 have successfully applied Pareto – concepts for gene selection coupled with the use of a series of parametric statistical methods 28. It is the intention of this work to keep the analysis strategy as nonparametric as possible, so as to not depend heavily on statistical assumptions or –in a different sense of nonparametric‐ the adjustment of parameters by the user that might bias the analysis results.

## Method: MCO Problem Formulation

**Figure 1:**
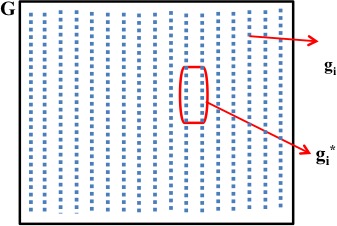
Problem representation where *G* = {*g*_i_}, *i *= 1,2,3,…,*n* and $g_{i}^{*}\in G$.

**Figure 2:**
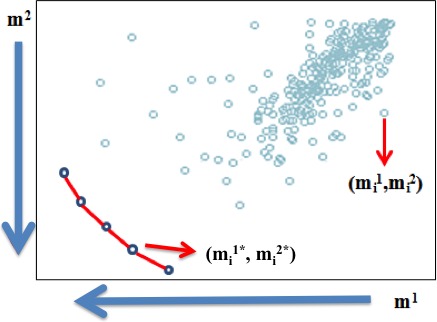
Representation of the Pareto‐efficient frontier of the MCO problem.

Figure 1 shows the elements of the graphical representation of the MCO problem. G denotes the universe of solutions that comprises the *n* genes to be analyzed with *g*_*i*_ representing each gene under analysis, (*i *= 1, 2, … *n*). Figure 2 shows the space defined by two criteria or PMs under analysis, *m* ^1^ and *m* ^2^. In the generalization of this figure, $m{}_{i}^{k}$ is the value for the *i*‐th gene in the *k*‐th PM. Then *k *= 1, 2, … *C,* where *C* is the number of PMs considered in the analysis. The Pareto‐efficient frontier in Figure 2 is formed by the genes $g{}_{i}^{*}$. These genes have indeed the best possible balances among the two PMs to be minimized and are the ones proposed as potential biomarkers.

When it comes to microarray analysis, the PMs of choice are usually related to the difference of gene expression measured in two distinct states for comparison purposes. Looking for the most differentially expressed genes is akin to looking for potential biomarkers, and it is a problem that can be casted as described up to this point.

According to Deb 29 and Ehrgott 30 the Pareto‐efficient solutions must meet the Pareto‐optimality conditions. In practical terms, this relates to finding nondominated solutions in the following sense: a solution X^(1)^ is said to dominate the other solution X^(2)^, if both conditions 1 and 2 are true:

1. The solution X^(1)^ is no worse than X^(2)^ in all PMs.
2. The solution X^(1)^ is strictly better than X^(2)^ in at least one PM.

These conditions can be evaluated for every single pair of genes to find those that are not dominated by any other gene. These are the Pareto‐efficient genes that form the Pareto‐efficient frontier of the MCO problem at hand.

As stated previously, in the search for the most differentially expressed genes, the expressions of all candidate genes are measured in two states to be then further compared. It is common, then, to use the difference of the means or the medians of the relative gene expression in these two states, for example. In this work, each of the C experiments will contribute one difference of medians between two states termed “control” and “cancer.” This translates into each gene being evaluated through C PMs. The absolute value of these differences will then be transformed to follow a minimization direction to match the illustration in Figure 2, where the following notation is introduced:

Let us represent the *i‐*th gene in terms of its values on each of the C PMs as $g_{i}\Leftrightarrow \left(m_{i}^{1},\;\;m_{i}^{2},\ldots ,\;m_{i}^{k},\ldots ,m_{i}^{C}\;\right)$ *,* for *i *= 1, 2, 3, …, *n*. Then, the objective of the analysis is to find the set of Pareto‐efficient solutions: $g_{i}^{*}\Leftrightarrow \left(m_{i}^{1*},\;\;m_{i}^{2*},\ldots ,\;m_{i}^{k*},\ldots ,\;m_{i}^{C*}\;\right)$. This is accomplished through a full pairwise comparison among the n genes as explained next.

First, a matrix δ^*k*^ is built for the *k*‐th PM resulting in *C* squared matrices of size *n* built as follows:

$$\delta^{k}=\begin{array}{cc} & \begin{array}{cccc} & m_{1}^{k} & m_{2}^{k} & \cdots \\ m_{1}^{k} & \delta {}_{11}^{k} & \delta {}_{12}^{k} & \cdots \\ m_{2}^{k} & \delta {}_{21}^{k} & \delta {}_{22}^{k} & \cdots \\ \vdots & \vdots & \vdots & \cdots \end{array}\begin{array}{c} m_{j}^{k}\\ \delta {}_{1j}^{k}\\ \delta {}_{2j}^{k}\\ \vdots \end{array}\begin{array}{c} \cdots \\ \cdots \\ \cdots \\ \cdots \end{array}\begin{array}{c} m{}_{n}^{k}\\ \delta {}_{1n}^{k}\\ \delta {}_{2n}^{k}\\ \vdots \end{array}\\ & \begin{array}{cccc} m{}_{i}^{k} & \delta {}_{i1}^{k} & \;\delta {}_{i2}^{k} & \cdots \end{array}\begin{array}{ccc} \;\delta {}_{1j}^{k} & \cdots & \delta {}_{in}^{k} \end{array}\\ & \begin{array}{cccc} \vdots & \;\;\;\;\;\vdots & \;\;\vdots & \;\cdots \end{array}\begin{array}{ccc} \;\vdots & \cdots & \vdots \end{array}\\ & \begin{array}{cccc} m{}_{n}^{k} & \delta {}_{n1}^{k} & \delta {}_{n2}^{k} & \cdots \end{array}\begin{array}{ccc} \;\delta {}_{nj}^{k} & \cdots & \delta {}_{nn}^{k} \end{array} \end{array}$$

where:

(1) $$\delta_{ij}^{k}=\left\{\begin{array}{c} -1,\;\;\text{if}\;m_{i}^{k}<m_{j}^{k}\\ 0,\;\;\text{if}\;m_{i}^{k}=m_{j}^{k}\\ W,\;\;\text{if}\;m_{i}^{k}<m_{j}^{k} \end{array}\;\text{for}\;i=1,\;\;2,\;\ldots ,\;n\right.j=\;\;1,\;\;2,\;\ldots ,\;\;n;\;k\;=1,\;\;2,\;\ldots ,C\;;$$

and *W* is defined as a large positive integer number used as a penalty. In this work, *W* = 1000 is used.

Next, a summation matrix is computed with elements $\alpha_{ij}=\sum_{k\;=\;1}^{C}\delta_{ij}^{k}\;$. This is exemplified in Table 1 when *C* = 2:

**Table 1:** All the possible combinations of a minimization problem for two criteria

Outcome number	$\delta_{ij}^{1}$	$\delta_{ij}^{2}$	*α*_*ij*_	Outcome
1	0	0	0	*X* ^*i*^ is not worse and not better either in *m* ^1^ or *m* ^2^
2	0	−1	−1	*X* ^*i*^ is better in *m* ^2^
3	0	W	W	*X* ^*i*^ is worse in *m* ^2^
4	−1	0	−1	*X* ^*i*^ is better in *m* ^1^
5	−1	−1	−2	*X* ^*i*^ is better in both *m* ^1^ and *m* ^2^
6	−1	W	W‐1	*X* ^*i*^ is better in *m* ^1^ and worse *m* ^2^
7	W	0	W	*X* ^*i*^ is worse in *m* ^1^
8	W	−1	W‐1	*X* ^*i*^ is better in *m* ^2^
9	W	W	2W	*X* ^*i*^ is worse in *m* ^1^ and *m* ^2^

A new matrix *γ* is then build by assessing the values *α*_*ij*_. For C=2, for example, the following assessment applies:

$$\gamma_{ij\;\;=\;\;}\left\{\begin{array}{c} W,\;\;if\;\;\;\;\alpha_{ij}\in \left\{0,\;W\right\}\\ 2W,\;\;if\;\;\;\;\alpha_{ij}=2W\\ 0,\;\;\;\text{otherwise} \end{array}\right.,\;\left\{\begin{array}{c} i=1,2,\ldots n\\ j=1,2,\ldots n \end{array}\right.$$

In general for any value *C* ≥ 2

(2) $$\;\;\gamma_{ij\;\;=}\left\{\begin{array}{c} \frac{C}{2}W,\;\;if\;\;\;\alpha_{ij}\in \left\{0,\;W,\;\ldots ,\left(C-1\right)W\right\}\;\\ CW,\;\;if\;\;\;\alpha_{ij}=CW\\ 0,\;\;\;\text{otherwise} \end{array}\right.,\left\{\begin{array}{c} i=1,\;\;2,\ldots n\\ j=1,\;\;2,\ldots n \end{array}\right.$$

Thus, in summary, this process will result in the *γ* matrix:

$$\gamma =\begin{array}{cc} & \begin{array}{cccc} & {m_{1}}^{\cdot } & {m_{2}}^{\cdot } & \cdots \\ m{}_{1}^{c} & \gamma_{11} & \gamma_{12} & \cdots \\ m{}_{2}^{c} & \gamma_{21} & \gamma_{22} & \cdots \\ \vdots & \vdots & \vdots & \cdots \end{array}\begin{array}{c} {m_{j}}^{\cdot }\\ \gamma_{1j}\\ \gamma_{2j}\\ \vdots \end{array}\begin{array}{c} \cdots \\ \cdots \\ \cdots \\ \cdots \end{array}\begin{array}{c} {m_{n}}^{\cdot }\\ \gamma_{1n}\\ \gamma_{2n}\\ \vdots \end{array}\\ & \begin{array}{cccc} m{}_{i}^{c} & \;\gamma_{i1} & \;\;\;\;\gamma_{i2} & \;\cdots \end{array}\begin{array}{ccc} \gamma_{ij} & \cdots & \gamma_{1n} \end{array}\\ & \begin{array}{cccc} \;\;\vdots & \;\;\;\;\;\vdots & \;\;\;\;\vdots & \;\;\;\;\cdots \end{array}\begin{array}{ccc} \;\;\vdots & \cdots & \vdots \end{array}\\ & \begin{array}{cccc} m{}_{n}^{c} & \gamma_{n1} & \;\;\gamma_{n2} & \;\;\cdots \end{array}\begin{array}{ccc} \gamma_{nj} & \cdots & \gamma_{nn} \end{array} \end{array}$$

In order to find $g{}_{i}^{*}$, a vector *β* is built containing the sums of each row of matrix *γ* as follows:

(3) $$\beta_{i}=\sum_{j\;=\;1}^{n}\gamma_{ij},\;\;i=1,\;2,\ldots n$$

$$\beta \;=\begin{array}{cc} & \begin{array}{cccc} \beta_{1} & = & \gamma_{11} & +\\ \beta_{2} & = & \gamma_{21} & +\\ \beta_{3} & = & \gamma_{31} & +\\ \vdots & & \vdots & \end{array}\begin{array}{cccc} \gamma_{12} & + & \ldots & \gamma_{1j}\\ \gamma_{22} & + & \ldots & \gamma_{2j}\\ \gamma_{32} & + & \ldots & \gamma_{3j}\\ \vdots & & \cdots & \vdots \end{array}\begin{array}{c} +\\ +\\ + \end{array}\begin{array}{c} \ldots \\ \ldots \\ \ldots \\ \cdots \end{array}\begin{array}{c} \gamma_{1n}\\ \gamma_{2n}\\ \gamma_{3n}\\ \vdots \end{array}\\ & \begin{array}{cccc} \beta_{i} & \;= & \gamma_{i1} & \;+ \end{array}\begin{array}{cccc} \gamma_{i2} & \;+ & \ldots & \gamma_{ij} \end{array}\begin{array}{ccc} + & \cdots & \gamma_{in} \end{array}\\ & \begin{array}{cccc} \vdots & & \;\;\;\;\;\;\;\;\vdots & \end{array}\begin{array}{cccc} \;\;\;\;\;\;\;\vdots & & \;\;\;\;\;\;\cdots & \;\;\vdots \end{array}\begin{array}{ccc} & \;\;\cdots & \vdots \end{array}\\ & \begin{array}{cccc} \beta_{n} & = & \gamma_{n1} & + \end{array}\begin{array}{cccc} \gamma_{n2} & + & \cdots & \gamma_{nj} \end{array}\begin{array}{ccc} + & \cdots & \gamma_{nn} \end{array} \end{array}$$

The Pareto‐efficient frontier will, then, contain all solutions that meet equation (4):

(4) $$g_{i}^{*}=\left\{g_{i}^{\;\;}|\;\beta_{i}<\text{CW},\;\;i=1,\;2,\ldots n\right\}$$

With this last step, the Pareto‐efficient solutions, $\;g_{i}^{*}=\left\{m_{i}^{1*\;\;}\right.,\left.m_{i}^{2*\;\;},\ldots m_{i}^{k*\;},\;\ldots ,\;m_{i}^{C*}\right\}$ *,* are clearly identified.

This algorithm identifies all the solutions of the Pareto‐efficient frontier. The maximum number proved and coded in this work is five PMs. The MatLab code is available in Appendix A1. In addition, Appendix A2 contrasts the proposed method with the use of a volcano plot to detect differentially expressed genes. Indeed, the mathematical description provided here is sufficient for the interested reader to code the method. The next illustration should help in this endeavor.

### Implementation of method

The next example will explain the application of the method. The objective is to find the Pareto‐efficient solutions $\;g_{i}^{*}\;$ for the minimization of two PMs (*C* = 2).

**Figure 3:**
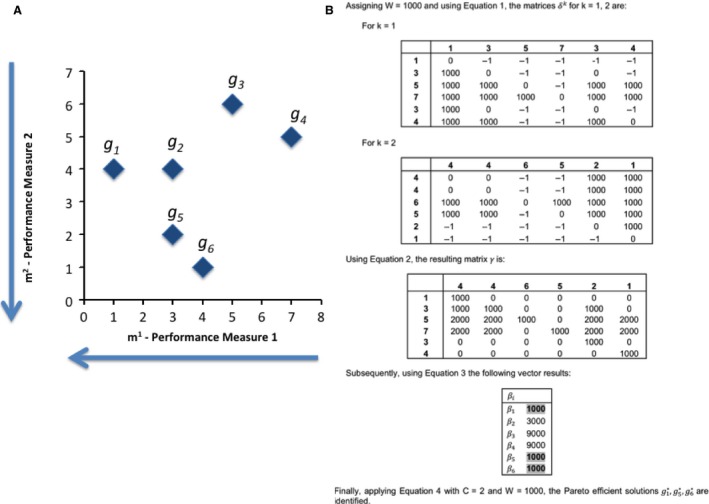
Graphical and Mathematical representation of the sample problem. (A) The six candidate solutions of the sample problem. (B) Mathematical formulation of the problem.

Let *G *= {*g*_1_, *g*_2_, *g*_3_, *g*_4_, *g*_5_, *g*_6_} be a set of *n* = 6 genes. The values for the PMs per gene are *g*_1_(1, 4)*; g*_2_(3,4)*; g*_3_(5,6)*; g*_4_(7,5)*; g*_5_(3,2)*; g*_6_(4,1). This leads to having $\left\{m{}_{1}^{1}m{}_{2}^{1}m{}_{3}^{1}m{}_{4}^{1}m{}_{5}^{1}m{}_{6}^{1}\right\}$ = {1, 3, 5, 7, 3, 4} and $\left\{m{}_{1}^{2}m{}_{2}^{2}m{}_{3}^{2}m{}_{4}^{2}m{}_{5}^{2}m{}_{6}^{2}\right\}$ = {4, 4, 6, 5, 2, 1}. Figure 3 shows the MCO problem for the case of minimization of both performance measures and its mathematical solution.

**Figure 4:**
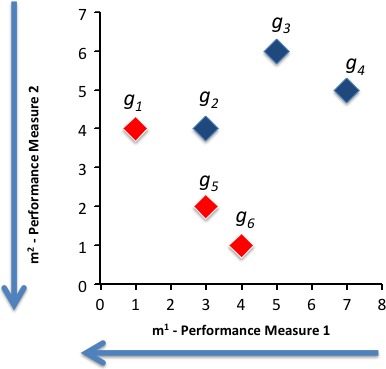
Pareto‐efficient solutions for the sample problem.

Finally, applying equation (4) the Pareto‐efficient solutions implies comparing the beta values to a threshold of 2000. The solutions $g_{i}^{*},\;$ for this MCO problem are $g_{1}^{*},g_{5}^{*},g_{6}^{*}$. These solutions are graphically shown in Figure 4.

## Analysis and Results of Lung cancer Microarray

**Figure 5:**
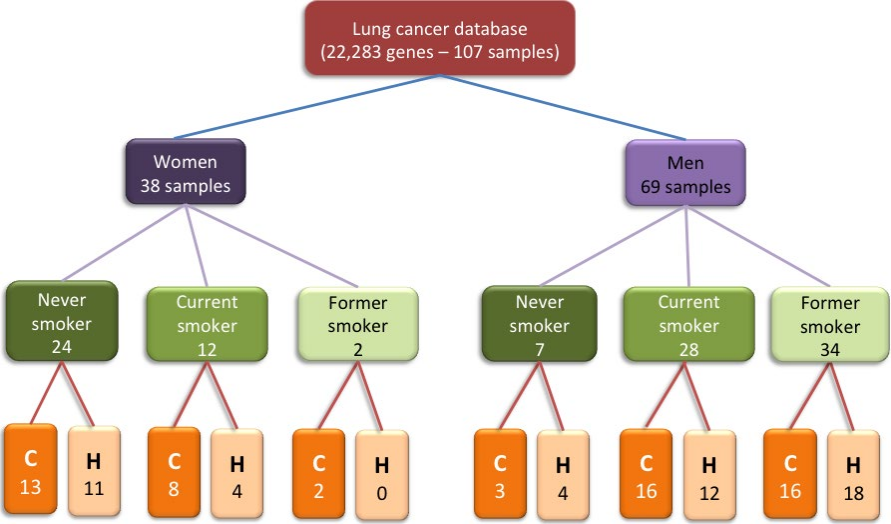
Organization of database GDS3257. “C” indicates cancer and “H” indicates controls.

In this analysis, the database with GEO identifier GDS3257 was used. This database was first reported by Landi MT and collaborators 31. The database contains measures of relative expression for 22,283 genes from 107 samples: 49 control and 58 cancerous tissues. The age of the donors was between 44 and 79 years old. Samples were from never smokers, former smokers, and current smokers (See Fig. 5).

### Case 1: Comparative analysis between different pairs of subgroups

**Figure 6:**
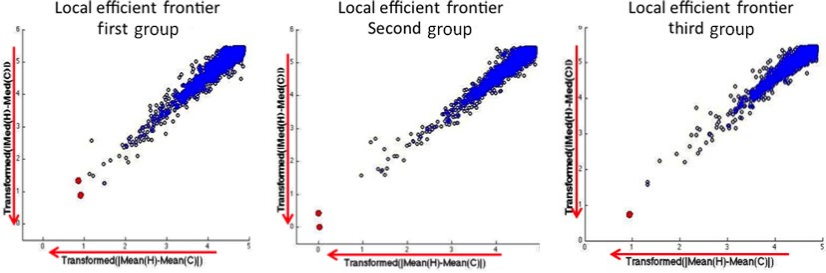
Local Pareto‐efficient frontiers of all groups. For the first and second groups, two genes are at the local Pareto‐efficient frontier, and only one gene for the third group.

**Figure 7:**
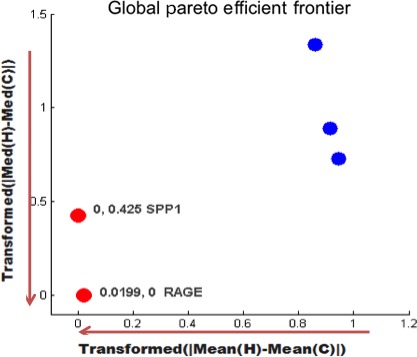
Globally‐optimal Pareto‐efficient frontier consisting of *RAGE* and *SPP1* genes.

For the first analysis the group of never smokers was considered and the comparison was between controls and cancer samples. There were fifteen controls (HNS) and sixteen cancer (CNS) samples. The absolute value of the differences of means and medians for each gene were calculated. The analysis in MatLab tool was run in a computer with 4 GB of memory RAM and 2.66 GHz CPU. Due to this memory constraint, the Pareto‐efficient frontier was found in a tournament fashion 32 as explained next. The 22,283 genes were divided into three groups: two groups of 7500 and one of 7283 genes. The MatLab tool was used to find the locally efficient frontier in each group. Finally, the genes in each one of the three efficient frontiers were analyzed together to find the global Pareto‐efficient frontier. It is important to point out that the order of the partition and input of the data does not affect the final efficient frontier, as this is a case of explicit full comparison. In one criterion, the process would be similar to finding the tallest person in a room by picking the tallest one in different subgroups and comparing the local winners in the end to find the global winner. With enough computing memory, partitioning the data is not necessary. For each group, the locally nondominated subset was identified (Fig. 6). Then the locally nondominated subsets were used to obtain the globally optimal Pareto‐efficient frontier, as seen in Figure 7. For this first analysis *RAGE* and *SPP1* are the genes in the global Pareto‐efficient frontier. It is important to recall that the user does not need to normalize or use a threshold value to achieve this result.

For the second analysis the selected group was the one for the current smokers, and again the comparison was between control current smokers (HCS) and cancer current smokers (CCS). The group had 16 samples for HCS and 24 samples for CCS. The process was performed as in the previous analysis. In this case the global Pareto‐efficient frontier had just one gene, the *SPP1*.

A third analysis compared groups HNS and CCS. There were 15 HNS samples and 24 CCS samples and *RAGE* was the only solution. In the fourth analysis comparing the 16 HCS samples and the 16 CNS samples the gene with the largest change is *SPP1*.

In a fifth analysis, the 15 HNS samples are compared with the 16 samples of HCS, resulting in three genes in the efficient frontier: *RPS4Y1*,* CYP1B1,* and *XIST*. When, in the sixth analysis, the comparison is done for the cancer group between nonsmokers (CNS, 16 samples) and current‐smokers (CCS, 24 samples) there is only one gene present in the solution: *XIST*.

**Figure 8:**
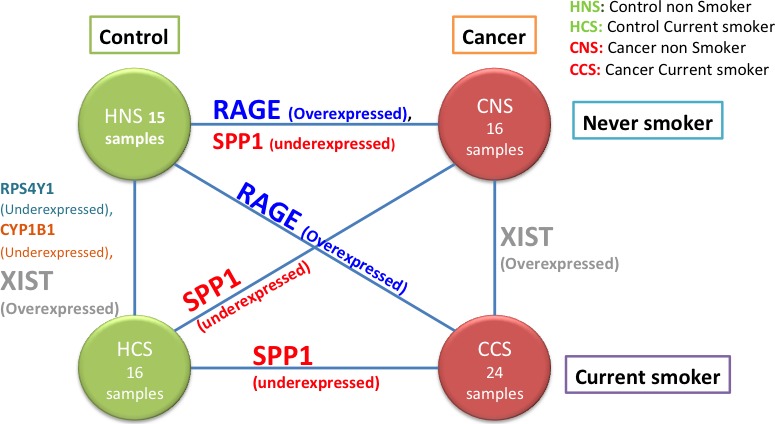
Diagram representing six analyses between four different conditions (HNS, HCS, CNS, CCS). The edges of the graph list the genes in the associated Pareto‐efficient frontier.

Figure 8 shows a summary of the six analyses between never smoker versus current smoker in cancer and control tissues. The circles on the left side represent the controls never smoker (HNS) and controls current smoker (HCS) tissues, while the circles on the right hand side represent the cancer never smoker (CNS) and cancer current smoker (CCS) tissues. Additionally, the upper circles represent never smoker tissues, whereas the lower circles symbolize current smoker tissues.

### Case 2: Analysis of lung cancer in women: never smoker versus current smoker in cancer and control tissues

**Figure 9:**
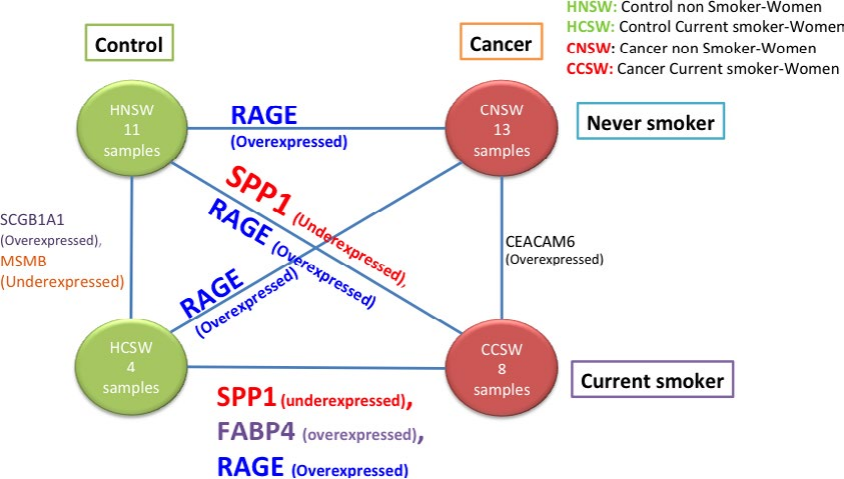
Diagram representing six analyses between four different conditions for women samples (HNSW, HCSW, CNSW, CCSW). The edges of the graph list the genes in the associated Pareto‐efficient frontier.

Figure 9 shows the result with the same analysis described before, but selecting only for women's tissues. For this representation, the only efficient solution is *RAGE*, which showed a large change when controls (HNS and HCS) were compared to cancer.

### Case3: Analysis of lung cancer in men: never smoker versus current smoker

**Figure 10:**
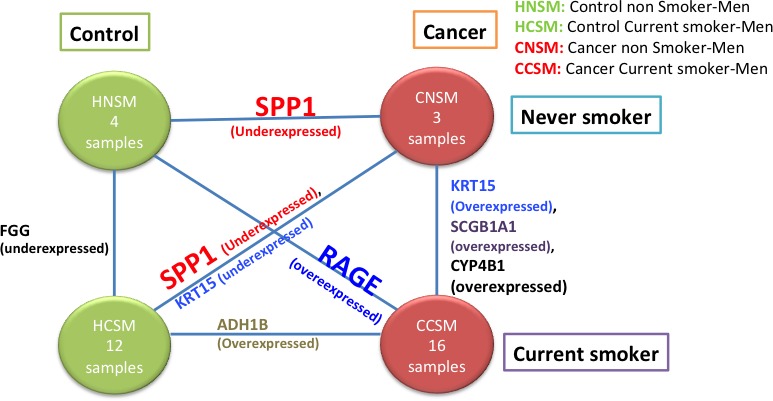
Diagram representing six analyses between four different conditions for men samples (HNSM, HCSM, CNSM, CCSM). The edges of the graph list the genes in the associated Pareto‐efficient frontier.

Figure 10 shows the results with an analysis similar to the one described before, but using only men samples. For this representation, as in previous cases, *RAGE* and *SPP1* showed significant changes when controls (HNS or HCS) were compared to cancer.

Table 2 shows the scientific names of genes obtained in the Pareto‐efficient frontier from all previous analyses.

**Table 2:** Scientific names the genes identified in the analyses of this work

Official symbol	Official name
RAGE	Receptor for Advanced Glycosylation End Products
SPP1	Secreted PhosphoProtein 1
XIST	X Inactive Specific Transcript (nonprotein coding)
RPS4Y1	Ribosomal Protein S4, Y‐linked 1
CYP1B1	Cytochrome P450, family 1, subfamily B, polypeptide 1
FABP4	Fatty Acid Binding Protein 4, adipocyte
CEACAM6	Carcinoembryonic Antigen‐related Cell Adhesion Molecule 6 (nonspecific cross reacting antigen)
MSMB	Microseminoprotein, beta
SCGB1A1	Secretoglobin, family 1A, member 1 (uteroglobin)
ADH1B	Alcohol Dehydrogenase 1B (class I), beta polypeptide
CYP4B1	Cytochrome P450, family 4, subfamily B, polypeptide 1
KRT15	Keratin 15
FGG	Fibrinogen Gamma chain

### Case 4: The possibility of meta‐analysis with four performance measures: a prototype for meta‐analysis

**Figure 11:**
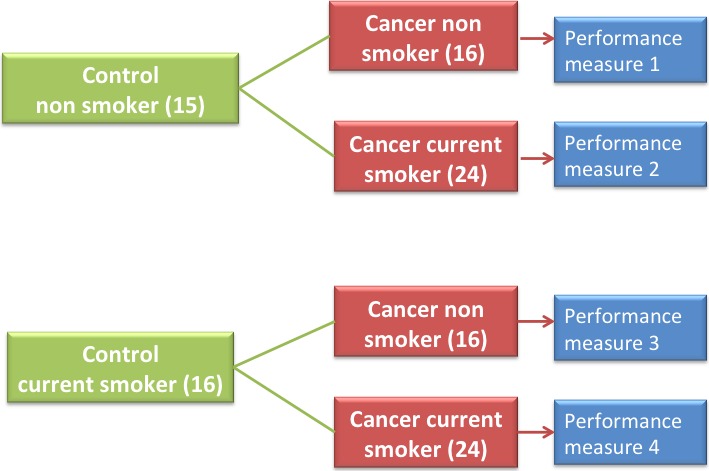
Groups for meta‐analysis with four PMs.

In the previous analyses two PMs (absolute value of differences in means and absolute value of differences in medians) were used. In this analysis, MCO meta‐analysis is carried out using four PMs, which were the absolute value of differences in medians for each group 16. The medians were used for their nonparametric characteristics, as it has been habitual in analyses previously carried out by our group. Continuing with the case, the difference in medians between the groups of cancer and control tissues is calculated for each one of the 22,283 genes in the database. These groups are: HNS (15 samples) versus CNS (16 samples), HNS (15 samples) versus CCS (24 samples), HCS (16 samples) versus CNS (16 samples), HCS (16 samples) versus CCS (24 samples) as seen in Figure 11.

In this way, the four PMs were calculated and MCO was applied to find the genes with high variation levels of the relative expressions throughout all PMs. Among all the 22,283 genes and using four PMs, the genes with high variation were *RAGE* and *SPP1*. This analysis supports the potential of the proposed method for meta‐analysis.

## Discussion

Table 3 presents the summary of genes obtained from eighteen analyses of the lung cancer database. The first group consists of the genes obtained from an analysis from both women and men. The second group is obtained from a group analysis of only women, and the last group is the results of a group analysis of only men. The common genes for all groups are *RAGE*,* SPP1*, and *SCGB1A1*. The products of these three genes are associated with inflammatory processes and different cancer types including lung 23, 34, 35, 36, 37, 38. From this table, three important conclusions are obtained. First, those genes found in the literature as biomarkers such as *CYPIB1* 39 and FABP4 40 validate our method. Secondly, those genes found in the literature as associated with other types of cancer, such as, *XIST* (a nonprotein coding gene) 41, among others, could eventually be validated and proposed as lung cancer biomarkers with the precursor that they are important genes for other types of cancer and could uncover relations between different cancer types. Also, these genes could possibly have a relation with lung cancer biomarkers in a pathway to be researched. Third, the genes that do not have any evidence found in literature indicating or any identification as biomarkers in other types of cancer, are the opportunities for discovery and thus, offer the potential for a larger contribution.

**Table 3:** Summary from Pareto‐efficient frontier genes and their related cancer

Gene name	Examples of cancer types where the gene is involved	Reference
RAGE	Pancreas, colon and prostate, colorectal, gastric, liver, lung	42, 43, 44, 45, 46, 47
SPP1	Oral, lung, bone, bladder, prostate, cervical, breast, head and neck, liver	36, 48, 49, 50, 51, 52, 53, 54
XIST	Meninges, breast, ovarian	55, 56, 57
RPS4Y1	Meninges	55
CYP1B1	Lung, cervical, head and neck, prostate	58, [59, p. 1], 60, [61, p. 1]
*Genes from the analysis with data pertaining only to Women*		
FABP4	Prostate and breast, ovarian	62, 63
MSMB	Prostate	64
CEACAM6	Head and neck, breast, colon, lung	65, 66, 67, 68
SCGB1A1*	Lung	69
*Genes from the analysis with data pertaining only to Men*		
FGG	Liver	70
KRT15	Lung, ovarian	71, 72
ADH1B	Esophageal, colorectal, head and neck	73, 74, 75
CYP4B1	Bladder	76
SCGB1A1*	Lung	69

## Conclusions

The method applied in this study could be used to analyze data related to biological health care research where microarrays and other –omics are the driving experiments for exploration.

The tool coded in MatLab can currently analyze five criteria, that is, it can be used to meta‐analyze up to five different datasets in one run. The discrimination rate makes the analysis very manageable. Also, the results will be friendly and conveniently available to physicians or biological researchers, as this analysis does not require normalization, preference of objectives, parameter adjustments by user, or the definition of a threshold value. Importantly, the mathematical treatment is easy to translate into a functional code of the analyst's choice.

In the case study in lung cancer the general conclusions are: *RAGE* and *SPP1* showed large change between controls and cancer. Moreover, *SPP1* showed a large change between the Control Current Smoker and the Cancer Nonsmoker, and *RAGE* showed large change between Control Never Smoker and Cancer Current Smoker. Also, *XIST* showed a large difference when comparing Never Smoker and Current Smoker (both in control and cancer tissues). The fact that these genes have already been related to cancer, indicate the capability of the proposed method.

It should be taken into consideration that *SCGB1A1* was found in this study to have an over expression in both Cancer Never Smoker and Cancer Current Smoker. However, *SCGB1A1* expression has been found to be reduced in current smokers 60 **.** Further biological studies should be performed to validate the results obtained by the methodology applied in this study.

Currently we are working on improving the usability of the code to make the method more amicable to the users. Future work should include further investigation of the potential biomarkers proposed in this document and experimental validation. It is certainly also envisioned the future tests of the proposed method with different –omics.

## Conflict of interest

None declared.

## Figures and Tables

**Figure A1:**
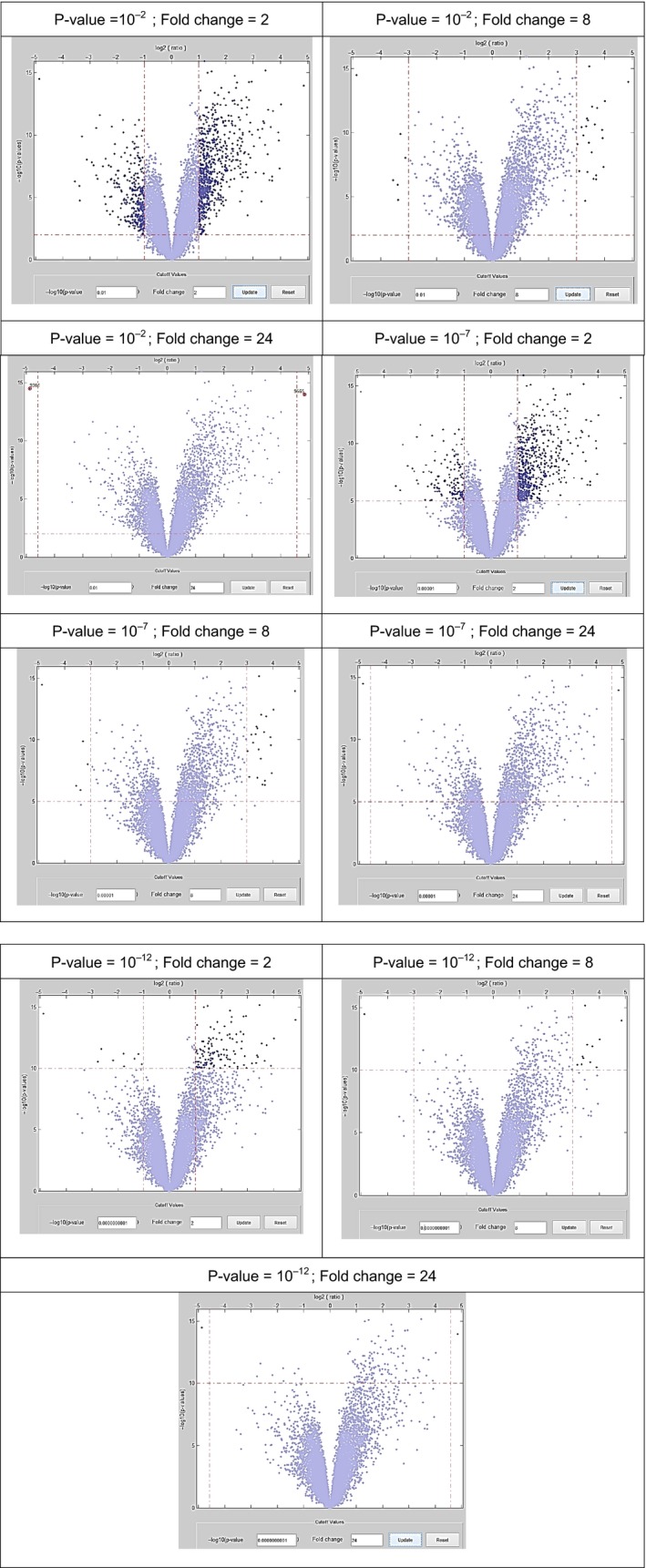
Figures represent the results of genes with high DE using Volcano plot and varying the *P*‐values and FC. The dark blue color indicates the genes most differentially expressed.

## Appendix A1 Matlab code to run the proposed MCO strategy

%Program: Analysis of Pareto frontier for five criteria

%Author: Katia I Camacho-Caceres

dataT = load("data5Criteria.txt"); %Load data

[x,y] = size(dataT);

data = dataT(:,2:end);

[n,m]=size(data);

c1 = 1000*ones(n,n,m); % matrix for first condition

for j=1:m

     for a=1:n

           for b=1:n

                 if data(a,j) == data(b,j)

                       c1(a,b,j)=0;

                 elseif data(a,j)<data(b,j)

                       c1(a,b,j)=-1;

                 end

           end

     end

end

% Sum two matrices

c2=zeros(n,n);

for a=1:n

for b=1:n

if c1(a,b,1)+c1(a,b,2)+c1(a,b,3)+c1(a,b,4)+c1(a,b,5)==0

c2(a,b)=2500;

elseif c1(a,b,1)+c1(a,b,2)+c1(a,b,3)+c1(a,b,4)+c1(a,b,5)==1000

c2(a,b)=2500;

elseif c1(a,b,1)+c1(a,b,2)+c1(a,b,3)+c1(a,b,4)+c1(a,b,5)==2000

c2(a,b)=2500;

elseif (c1(a,b,1)+c1(a,b,2)+c1(a,b,3)+c1(a,b,4)+c1(a,b,5))==3000

c2(a,b)=2500;

elseif (c1(a,b,1)+c1(a,b,2)+c1(a,b,3)+c1(a,b,4)+c1(a,b,5))==4000

c2(a,b)=2500;

elseif (c1(a,b,1)+c1(a,b,2)+c1(a,b,3)+c1(a,b,4)+c1(a,b,5))==5000

c2(a,b)=5000;

end

end

end

% Find non-dominated set (ncd), and dominated set (cd)

cnd = zeros(x,y);

cd = zeros(x,y);

i=0;

j=0;

for a=1:x

sumfila=sum(c2(a,:));
	 if sumfila>=5000; % condition for dominated set

i=i+1;

cd(i,:)=dataT(a,:);

else % non-dominated set

j=j+1;

cnd(j,:)=dataT(a,:);

end

end

index = 1:x;

disp([round(index") cd]);

disp([round(index") cnd]);

%Show non-dominated set in notepad file

%Position of gene,f1 ,f2 ,f3 ,f4 ,f5 for each criteria

disp(' Non-dominated set ');

cnd=cnd(1:j,:);

filecnd = fopen('cnd5CriteriaBio.txt","w");

fprintf(filecnd,"%6s %12s %12s %12s %12s %12s\r\n","Posicion","F1","F2","F3", 'F4", 'F5");

fprintf(filecnd,"%6.4f %12.4f %12.4f %12.4f %12.4f %12.4f\r\n",cnd");

fclose(filecnd);

## Appendix A2 MCO compared to the use of a volcano plot

### Volcano plot

In the literature there are many methods to detect DE genes from microarrays comparing two states. One of those methods is the Volcano Plot, which is a graphic method widely used by scientists and biologists 77. This method is implemented in different software packages. The MCO method proposed in this research is here compared to the volcano plot in a series of analysis.

Volcano plot is a scatter plot built using p‐values versus gene expression ratios of fold change (FC). This scatter plot used the negative log10‐transformed p‐values from the gene specific t‐test against the log2 fold change. Genes with statistically significant differential expression according to the gene‐specific t‐test will lie above a horizontal threshold line. Genes with large fold‐change values will lie outside a pair of vertical threshold lines 78.

*P‐values* were calculated by unpaired t‐test using the gene expression values from two experimental conditions: healthy and cancer tissues.

*Fold Chang*e is calculated as the ratio of the mean control and mean treatment observations. This is the extension of the difference of the logarithm of the control mean (*y*_1_) and the logarithm of the control treatment (*y*_2_):

$$\text{FC}=\text{log}\left(\bar{y_{1}}\right)-\text{log}(\left(\bar{y_{2}}\right)$$

The ordinary *t*‐statistic selects genes with low standard deviations while the fold‐changes select genes with large shifts between control and treatment. Since the fold‐changes and the ordinary *t*‐statistic select different sets of genes, a researcher must decide whether a gene's importance is best quantified by the shift in expression or by the shift relative to the standard deviation.

According to the literature on the use of volcano plot, a researcher should choose the measure of differential expression based on the biological system of interest. On the one hand, if large absolute changes in expression are relevant to the system, then fold‐change should be used; on the other hand, if changes in expression relative to the underlying noise are important, then a modified t‐statistic is preferable. This, however, is the point of view from which this thesis wants to depart: the choice of ad‐hoc threshold values to select genes.

The analysis is required to choose threshold values for both measures to select important genes. The volcano plot is available in the bioinformatics toolbox for MatLab.

Given a particular microarray set with genetic expression levels measured. In two distinct states, the tool in MatLab obtains a p‐value per gene using a t‐test, and measures the FC in a logarithmic scale with base 2.

### Comparison of volcano method using lung cancer microarray

The original database GDS3257 of lung cancer was used for this analysis. The samples HNS and CNS were used to build the Volcano plot. As mentioned previously, the Volcano plot requires the user to define thresholds for two parameters: p‐value and FC to select genes. A 3^2^ factorial experiment was used to explore these parameters as shown in Figure A1. The results are shown in Table A1.

From Table A1, it can be seen how the results depend highly in the user's selection of thresholds. The combinations that exactly match the output of MCO in this instance are the ones with FC = 24 at any of the values chosen for *P*‐value in this experiment.

**Table A1:** Summary of important genes expressed using volcano plot

P‐value	Fold change	Differential expression	Overexpressed	Underexpressed
10^−2^	2	934	645	289
10^−2^	8	29	23	6
10^−2^	24	2	1	1
10^−7^	2	649	516	133
10^−7^	8	27	22	5
10^−7^	24	2	1	1
10^−12^	2	130	121	9
10^−12^	8	12	11	1
10^−12^	24	2	1	1
